# [Retracted] MicroRNA‑212 inhibits the metastasis of nasopharyngeal carcinoma by targeting SOX4

**DOI:** 10.3892/or.2023.8570

**Published:** 2023-05-15

**Authors:** Chengyi Jiang, Hongtao Wang, Lei Zhou, Tao Jiang, Yajia Xu, Lin Xia

Oncol Rep 38: 82–88, 2017; DOI: 10.3892/or.2017.5641

Following the publication of this paper, it was drawn to the Editor's attention by a concerned reader that certain of the cell migration and invasion images shown in Fig. 6B, C, E and F were strikingly similar to data that had already appeared in other articles. Owing to the fact that the contentious data in the above article had already been published prior to its submission to *Oncology Reports*, the Editor has decided that this paper should be retracted from the Journal. The authors were asked for an explanation to account for these concerns, but the Editorial Office did not receive a reply. The Editor apologizes to the readership for any inconvenience caused.

